# Individual- and Organization-Level Work-to-Family Spillover Are Uniquely Associated with Hotel Managers' Work Exhaustion and Satisfaction

**DOI:** 10.3389/fpsyg.2016.01180

**Published:** 2016-08-17

**Authors:** Soomi Lee, Kelly D. Davis, Claudia Neuendorf, Alicia Grandey, Chun Bun Lam, David M. Almeida

**Affiliations:** ^1^Department of Biobehavioral Health, Pennsylvania State UniversityUniversity Park, PA, USA; ^2^School of Social and Behavioral Health Sciences, Oregon State UniversityCorvallis, OR, USA; ^3^Institute for Educational Quality Improvement, Humboldt-Universität ZuBerlin, Germany; ^4^Department of Psychology, Pennsylvania State UniversityUniversity Park, PA, USA; ^5^Department of Early Childhood Education, The Education University of Hong KongHong Kong, China; ^6^Department of Human Development and Family Studies, Pennsylvania State UniversityUniversity Park, PA, USA

**Keywords:** conservation of resources theory, emotional exhaustion, hotel industry, job satisfaction, organizational climate, work-to-family spillover

## Abstract

**Purpose:** Building on the Conservation of Resources theory, this paper examined the unique and interactive associations of negative and positive work-to-family spillover (NWFS and PWFS, respectively) at the individual and organizational level with hotel managers' work exhaustion and satisfaction, beyond job demands and supervisors' leadership style.

**Design/Methodology/Approach:** Guided by the levels of analysis framework, we first tested the unique associations of NWFS and PWFS with emotional exhaustion and job satisfaction at the individual level (571 hotel managers), beyond job demands supervisors' leadership style. Second, using multilevel modeling, we tested the climate effects of NWFS and PWFS on emotional exhaustion and job satisfaction aggregated at the organizational level (41 hotels). Third, we examined the role of the organizational climate of PWFS in the associations of individual-level NWFS with emotional exhaustion and job satisfaction.

**Findings:** Beyond the effects of psychological job demands and supervisor's transformational leadership, at the individual level, hotel managers who experienced higher NWFS than other managers reported more exhaustion and lower job satisfaction, whereas those with higher PWFS reported less exhaustion and higher satisfaction. At the organizational level, working in hotels where the average level of NWFS was higher than other hotels was associated with feeling more exhaustion of the individual members; working in hotels with higher PWFS was associated with feeling less exhaustion. The negative link between individual-level NWFS and job satisfaction was buffered when organization-level PWFS was higher, compared to when it was lower.

**Originality/Value:** This study moves beyond a focus on traditional job characteristics, toward considering individual and organizational experiences in the work-family interface as unique predictors of work exhaustion and satisfaction. Strengths of the study include illuminating organizational work-family climate effects such that coworkers' shared experiences of NWFS and PWFS explain individual members' work exhaustion, beyond their own experiences of spillover. The results also highlight that a high level of organizational PWFS can buffer the negative effects of individual NWFS.

## Introduction

Work and family are interconnected spheres of life that play a vital role in employee well-being. Work-to-family spillover (WFS) refers to the process whereby behaviors, moods, and stress from the work sphere affect those in the family sphere (Mennino et al., 2005). Negative WFS (NWFS) refers to strain from the workplace interfering with one's family and personal life, whereas positive WFS (PWFS) indicates positive moods and energy from work facilitating performance of family and personal roles (Grzywacz and Marks, 2000). Guided by the Conservation of Resources (COR) theory (Hobfòll, 1989, 2001) that links between resources and stress in the work-family interface, this study examined the unique and interactive associations of NWFS and PWFS with hotel managers' work-related emotional exhaustion and job satisfaction at the individual and organizational levels.

The U.S. hotel industry is a part of the service sector that provides 24/7 customer service. Examining hotel managers was of our interest, because their work experiences are more likely to be spilled over to the home because of their long work hours, unpredictable work schedules, and permeable boundaries between work and family (Cleveland et al., 2007; Lawson et al., 2013). During the pilot work in which this study is based, the hotel managers described frustrations that arise due to interruptions at home and having to be available at all times for hotel needs (Cleveland et al., 2007), which were associated with the experience of high NWFS (Lawson et al., 2013).

For hotel managers, emotional exhaustion and job satisfaction are two important work-related outcomes. Their service work requires emotional labor (Hochschild, 1983) that may have emotional consequences (Wharton, 1993). Emotional exhaustion refers to a state of fatigue and burnout in response to one's work (Maslach and Jackson, 1981), and it indicates a problem in the work context that needs addressing to avoid both health and performance issues. A high level of emotional exhaustion is associated with poor physical health (Kim et al., 2011), the prevalence of early retirement intentions (von Bonsdorff et al., 2010), and low job performance (Halbesleben and Bowler, 2007). Job satisfaction is one of the most studied variables in organizational psychology that indicates the extent of positive attitudes about the job (Kashefi, 2009). Given the amount of time spent at work and its importance in providing meaning to employees' lives, it is important to understand what enhances or diminishes job satisfaction. A high level of job satisfaction is also beneficial for organizations as it is associated with high organizational commitment and job performance, and low turnover rate and absenteeism (Harter et al., 2002). By assessing emotional exhaustion and job satisfaction, we sought to examine whether and how NWFS and PWFS predict important features of employee outcomes that are implicated in workplace productivity.

This study had three specific aims. The first aim was to test whether NFWS and PWFS were uniquely associated with hotel managers' work exhaustion and satisfaction, beyond job characteristics (i.e., psychological job demands and supervisor's transformational leadership). Hotel managers tend to have high psychological job demands (Cleveland et al., 2007), which have been strongly associated with high emotional exhaustion and low job satisfaction across occupations (Maslach et al., 2001; Lewig and Dollard, 2003). Hotel managers are also profoundly affected by their supervisors' behaviors because they need to get effective support and supervision to deal with demands from customers (Cleveland et al., 2007; Brownell, 2010). Supervisor's transformational leadership has been found to be important for hotel managers' emotional exhaustion and job satisfaction (Gill et al., 2006; Erkutlu, 2008; Clark et al., 2009). In relation with WFS, the majority of previous studies have examined the mediating role of WFS in the links between job characteristics and employee outcomes (Demerouti et al., 2005; Baeriswyl et al., 2016). However, according to the COR theory (Hobfòll, 1989), WFS reflects the interference with or facilitation of resource accumulation in the work-family interface, meaning more than characteristics of the job (i.e., job demands and transformational leadership). Thus WFS may explain unique variance in employee outcomes, rather than solely explaining a partial variance in the causal chain of job characteristics to employee outcomes (Demerouti et al., 2004). We also examine the unique importance of PWFS that is often neglected in prior research (Bianchi et al., 2010).

The second aim was to test the organizational climate effects of WFS on hotel managers' work exhaustion and satisfaction. Most research to date remains at the individual-level analysis, which lacks the ability to consider organizational influences (Bliese and Jex, 2002; Grzywacz et al., 2007; van Emmerik and Peeters, 2009). To address this gap, this study adopts the Kozlowski and Klein's (2000) levels of analysis framework that emphasized the importance of considering organizational factors at higher levels for individual outcomes at lower levels. Specifically, we investigate the organizational effects of NWFS and PWFS, using the aggregate of individual ratings at the same hotel, which reflects shared experiences of NWFS or PWFS between coworkers. The hotel industry is an appropriate field to study organizational climate effects, as individual hotels are separate units that are comparable to one another in their basic structure.

Lastly, the third aim was to examine the interactive associations of organizational PWFS and individual NWFS with hotel managers' work exhaustion and satisfaction. This type of cross-level interaction has been rarely examined in prior research, but it can test whether organization-level PWFS plays an important role as social support in the workplace, similar to the role of a high-level community support from the COR framework (Hobfòll, 2001). Findings from this study may contribute to the design of future workplace interventions that target to change work and family interconnections through informal support and formal policies that can decrease NWFS and increase PWFS.

## Theoretical background linking work-to-family spillover to emotional exhaustion and job satisfaction

In his COR theory, Hobfòll (1989, 2001) suggested that individual resources are vulnerable to stressful circumstances. Resources include time, energy, and conditions (e.g., family relationships) that are valued by the individual. The main tenet of the COR is that a loss or the potential loss of resources are psychologically threatening. When confronted with externally induced stressors, individuals strive to minimize net loss of resources (“conservation of resources”). When freed from stressors, individuals strive to develop a resource surplus in order to offset the possibility of future loss. Applied to our study, NWFS reflects one's loss of resources (e.g., less time and energy for the family) due to work stressors carried over to the home domain interfere with performing family roles. A loss of resources are stressful (Hobfòll, 1989), and thus experiencing higher NWFS may be associated with feeling higher emotional exhaustion and lower satisfaction toward work. In contrast, PWFS refers to a state of resource surplus (e.g., better mood and more energy for the family), such that positive experiences from work facilitate performing family roles. A gain of resources benefits one's emotional energy and motivation toward work, which may be manifested through low emotional exhaustion and high job satisfaction.

The effects of WFS on hotel managers' work exhaustion and satisfaction may exist beyond the effects of traditional indicators of job characteristics, such as job demands (Bakker and Demerouti, 2007) and supervisor's leadership style (Gill et al., 2006). For example, a mother who works as a hotel manager can experience high NWFS if emotional work at her job makes her less engaged in interactions with her child at home. The experience of high NWFS may make her less satisfied with her job, beyond how many demands (and also transformational leadership) she has on the job. Because NWFS and PWFS represent one's loss or gain of perceived resources in the work-family interface (Hobfòll, 1989), their influences on employee outcomes would be above and beyond job characteristics. Also note that high NWFS may not be translated to low PWFS: NWFS and PWFS are each important in their own right and the association between PWFS and employee outcomes is independent of NWFS (Grzywacz, 2000). We tested the associations of NWFS and PWFS with hotel managers' emotional exhaustion and job satisfaction, net of job demands and supervisor's leadership. Our first set of hypotheses is:

*H1. Individual-level NWFS is positively associated with hotel managers' emotional exhaustion, whereas individual-level PWFS is negatively associated with emotional exhaustion*.

*H2. Individual-level NWFS is negatively associated with hotel managers' job satisfaction, whereas individual-level PWFS is positively associated with job satisfaction*.

## Organizational work-to-family spillover on hotel managers' work exhaustion and satisfaction

In addition to individual WFS, we examined organizational WFS, by adopting the Kozlowski and Klein's (2000) levels of analysis framework that emphasized the importance of considering organizational factors at higher levels for individual outcomes at lower levels. Individual experiences of WFS aggregated at the organizational level—organizational WFS—represents shared experiences between coworkers through affective and behavioral sharing processes (Kelly and Barsade, 2001). Organizational NWFS may be perpetuated by hearing coworkers complain about lack of family time or having fewer coworkers available to pick up and cover for employees when they need help. Thus, organizational NWFS reflects a negative work-family climate, operationalized as the degree to which managers in a given hotel share similar experiences of work stress carrying over to the home and interfering with family responsibilities. In a similar way, organizational PWFS reflects a positive work-family climate, operationalized as the degree to which managers share similar experiences of positive work events carrying over to the home and facilitating family roles. Importantly, organizational climate of WFS may be associated with individual members' work outcomes beyond their own experience of WFS. Affective sharing process involves tuning in to the emotions and attitudes of others, affecting not only employees who have similar experiences, but also those who do not. There is some evidence of how affective and behavioral sharing between coworkers influence individual employees beyond their own experiences. O'Neill et al. (2009) reported that higher time demands and lower manager support about family needs aggregated at the organization-level (i.e., a negative climate) were linked to higher turnover intentions and lower organizational commitment of employees in the organization, beyond the effects of the individual-level reports. Feierabend et al. (2011) also showed that a positive work-family climate and dialogue about family issues in organizations had effects on high commitment to the organization and low intention to quit of employees *without* any child or elderly care responsibilities. Their findings suggest that individual-level experiences of NWFS or PWFS can be shared with coworkers which, in turn, may influence hotel managers who do not even directly experience such spillover effects.

To represent a shared experience between coworkers in the same organization, this study takes the direct consensus approach that creates the average of individual-level managers' reports within each hotel. Scholars generally agree that work-family research needs to move beyond the individual as the unit of analysis (Grzywacz et al., 2007; Spell and Arnold, 2007; van Emmerik and Peeters, 2009; Moen et al., 2015); however, there is no consensus on the operationalization of organization-level constructs. Some studies used the percentage of employees falling in the high-risk group per team (Bakker et al., 2006; van Emmerik and Peeters, 2009), while others used the aggregate of individual responses, called the direct consensus approach (Bliese and Jex, 2002; Spell and Arnold, 2007; Lam et al., 2010). In the direct consensus approach, organizations with a high average level of NWFS will have many individual members who experience high NWFS. Working in a hotel where the organization-level NWFS is high may have negative effects on the individual managers' outcomes, because their coworkers experience high demands and thus have lack of psychological resources available to others (e.g., less sensitive to others' pain), according to the COR perspective (Hobfòll, 1989). Organizations with the average level of PWFS is high will have many individual members who experience high PWFS. These individuals may give aid to coworkers who do not experience such high PWFS by sharing their own strategies (that worked for them) for how work can positively influence family life. The COR theory predicts that individuals enrich their resources by investing to other network resources with a long-term outlook, such as providing support to kith and kin (Hobfòll, 1989). Similarly, individual members with higher PWFS may give support to others in their work context to get help when they need it. In this sense, organizational PWFS may indicate the extent of social support by coworkers, such as role modeling behavior and practical as well as emotional support. Working in a hotel where the organization-level PWFS is high may have positive effects on the individual managers' outcomes, owing to the availability of coworker social support individual manager can draw upon (Hobfòll, 1989). To capture organizational climate effects of WFS that are independent of individual experiences of WFS, we controlled for individual WFS in our analyses. As such, we tested the organizational-level associations of WFS with hotel managers' work exhaustion and satisfaction, beyond the effects of individual-level WFS as well as job demands and supervisor's transformational leadership. We hypothesized:

*H3. Organization-level NWFS is positively associated with individual members' emotional exhaustion, after controlling for individual-level NWFS. In contrast, organization-level PWFS is negatively associated with individual members' emotional exhaustion, after controlling for individual-level PWFS*.

*H4. Organization-level NWFS is negatively associated with individual members' job satisfaction, after controlling for individual-level NWFS. In contrast, organization-level PWFS is positively associated with individual members' job satisfaction, after controlling for individual-level PWFS*.

## Interactive effects of organizational spillover and individual spillover on hotel managers' work exhaustion and satisfaction

Hobfòll (2001) highlighted that the influence of higher-level support, such as community- and organization-level support, becomes more important when individuals are under a lack of resources. More specifically, when individuals who have a lack of resources are surrounded by others who can provide social support, the negative effects of a lack of resources may be buffered. Linking this perspective to the current study, organization-level PWFS may buffer the negative effects of individual-level NWFS on hotel managers' work exhaustion and satisfaction. For example, managers who experience high levels of NWFS at the individual level may be less satisfied with their work. However, if they work in hotels where the average level of PWFS is high, the potential adverse effects of higher NWFS on emotional exhaustion and job satisfaction may become weaker, because they can take advantage of resources available from coworkers (Hobfòll, 1989).

This study is one of the first to examine the role of organizational PWFS, as coworker social support in the workplace. Many studies have examined the role of social support directly measured by individual employees' responses. Social support is closely related to overall emotional support (e.g., advice, listing), whereas organizational PWFS represents role modeling or advice between coworkers that is specific to the spillover process. In other words, working in an organization where many coworkers—who are “similar to me”—have positive work-family experiences indirectly shows the employee that such positive spillover is possible and offers hope and optimism (Hobfòll, 2001). Thus organizational PWFS assesses the availability of coworker social support specific to spillover, although it is an indirect measure compared to the social support measure. Our measure of organizational PWFS also has methodological strength. Responses on the social support measure can be biased in that emotionally exhausted or less satisfied employees may report less social support (Podsakoff et al., 2003). Organizational PWFS may have less self-report bias, by aggregating the individual members' responses at the organizational level.

To date, no previous studies have examined the cross-level interaction between organization-level PWFS and individual-level NWFS. However, a rare example of this kind can be seen in a study by Bliese and Castro (2000). They examined a three-way multilevel interaction between work demands, role clarity, and work-group level supervisory support predicting employees' psychological strain. They found that the negative effect of work demands on psychological strain was moderated by role clarity only when work-group support was high. This finding lends support to Hobfòll (2001) in that the importance of organization-level support became apparent for employees who experienced work overload and thereby needed role clarity. Extending upon the prior work, we tested the interaction effects between organization-level PWFS and individual-level NWFS on hotel managers' work exhaustion and satisfaction. In a similar vein, if individuals who experience high NWFS work in an organization where the average level of NWFS is also high, then it may exacerbate the adverse effect of their own NWFS due to contagion and no way to conserve. Although the COR theory does not explicitly predict that a lack of higher-level resources exacerbates individual-level strain, we tested whether organization-level NWFS interacts with individual-level NWFS to influence hotel managers' work exhaustion and satisfaction. Our last set of hypotheses is:

*H5. The positive association between individual-level NWFS and emotional exhaustion is weaker when organization-level PWFS is high compared to when it is low*.

*H6. The positive association between individual-level NWFS and emotional exhaustion is stronger when organization-level NWFS is high compared to when it is low*.

*H7. The negative association between individual-level NWFS and job satisfaction is weaker when organization-level PWFS is high compared to when it is low*.

*H8. The negative association between individual-level NWFS and job satisfaction is stronger when organization-level NWFS is high compared to when it is low*.

## Methods

### Participants and procedures

Data came from a project investigating connections between work stress, health, and family relations of employees in the hotel industry. Considering that increasing sample size at organization-level is critical to achieve high levels of statistical power in multilevel analyses (Scherbaum and Ferreter, 2008), the project investigators targeted to sample more than 40 hotels. The research design involved initial contact with General Managers (GMs) at each of 56 full-service hotels located across major U.S. regions. During the meeting with the hotel GMs, researchers asked for permission and access to managers at the hotel. Middle-level managers were contacted across all departments, including sales and marketing, human resources, room operations, and food and beverage operations. Approximately 83% of the managers who were contacted agreed to complete an interview (588 managers at 50 hotels). Structured telephone surveys were conducted by trained survey research center personnel using computer-assisted telephone interviewing procedures. Interviewers rephrased questions if participants did not understand the wording without altering the meaning. Participants received a $20 honorarium. This research was conducted following ethics outlined by the Institutional Review Board.

After excluding nine hotels that did not contribute at least three managers (for rationale see Krasikova and LeBreton, 2012), the final sample consisted of 571 managers at 41 hotels. On average per hotel, 14 managers provided data (*Range* = 3–53). Our sampling strategy met the suggested criteria by Scherbaum and Ferreter (2008) that sampling approximately 10 employees in 35 groups may achieve sufficient statistical power in organizational research.

The managers worked 12.97 years (*SD* = 7.88) in the hotel industry and 4.72 years (*SD* = 5.28) in their current hotel. The average age of the managers was 37.72 years (*SD* = 9.08), and the average length of their formal education was 15.25 years (*SD* = 1.70) which corresponds to 3 years of college, vocational or technical school. About half of them (51.49%) were men. More than half (64.97%) were married or cohabitating with a permanent romantic partner. Of those managers who had a partner, the partner was employed in 81.94% of the cases. The average number of biological or adopted children living in the household was 0.81 (*SD* = 1.11) and 13.31% of the managers had young children aged 0–7.

### Measures

#### Psychological job demands

We used the job demands subscale by Karasek et al. (1998), which measures psychological stressors such as time pressure and workload. The job demands scale consists of seven items such as, “Your job requires your working fast” and “Your job requires a great deal of work to be done.” The responses ranged from 1 (strongly disagree) to 4 (strongly agree). Cronbach's α was 0.78.

#### Supervisor's transformational leadership style

Managers reported about leadership styles of GMs who were their direct supervisors. There was only one GM at each hotel, and several managers within a hotel reported about the same GM (supervisor). Transformational leadership style was measured by fifteen items from the Multifactor Leadership Questionnaire (MLQ 5X; Bass and Avolio, 1995). The 15 items comprise of four sub-factors of transformational leadership: idealized influence attributed and behavior (6 items), inspirational motivation (3 items), intellectual stimulation (3 items), and individualized consideration (3 items). We used the mean of the fifteen items. An example item (in idealized influence) is, “Your supervisor instills pride in you for being associated with him/her.” Each response ranged from 1 (not at all) to 5 (frequently, if not always). Cronbach's α was 0.93.

#### Negative and positive work-to-family spillover

We used work-to-family spillover scales from the National Survey of Midlife Development in the United States (Grzywacz and Marks, 2000). NWFS was measured by four items including, “Your job reduces the effort you can give to activities at home.” PWFS was assessed by asking three items such as, “The things you do at work help you deal with personal and practical issues at home.” The original PWFS scale consists of four items, but we removed one item (“having a good day on your job makes you a better companion when you get home”) due to low correlations with the other three items. Each response ranged from 1 (never) to 5 (all the time). Cronbach's α of NWFS was 0.80 and α of PWFS was 0.61. A confirmatory factor analysis with the seven items of WFS corroborated that there are two factors that clearly distinguish three items of PWFS from the four items of NWFS.

#### Emotional exhaustion

Wharton's (1993) Job-Related Exhaustion Scale was used to capture how tired or stressed out managers feel at the end of an average work day. The scale consists of five items, such as, “You feel used up at the end of the work day.” Responses ranged from 1 (strongly disagree) to 4 (strongly agree). Cronbach's α was 0.90.

#### Job satisfaction

Three items from a scale by Kopelman et al. (1983) were used. Kopelman et al. (1983) modified the 3-item General Job Satisfaction scale that is part of the Job Diagnostic Survey (Hackman and Oldham, 1975). The modification was substituting the term “career” for the word “job.” A sample item is, “You are satisfied with your present job situation.” Each response ranged from 1 (strongly disagree) to 5 (strongly agree). Cronbach's α was 0.81.

#### Covariates

We considered managers' individual and family characteristics that may denote degrees of family-related demands and resources and thus may relate to their work exhaustion and satisfaction. Those include managers' age (in years), gender (0 = female, 1 = male), number of children, and the presence of young children aged 0–7 (0 = no, 1 = yes). Given that living with a non-working spouse/partner may help the managers in reducing tensions between work and family roles, partner employment status was categorized as living with an unemployed partner (0) or employed partner (1) versus being single (2; the reference category). As such, we combined married/partnered status and partner's employment status into one variable, because most spouses/partners were employed (81.94%) and thus including two variables was redundant. We also controlled for negative affect that occurred in the past 2 weeks. In this way we are tapping into more trait like aspects of negative affect. We used the mean across 10 items (“To what extent you have felt: scared, afraid, upset, distressed, jittery, nervous, ashamed, guilty, irritable, and hostile, in the past 2 weeks?”) from the Positive and Negative Affect Schedule (PANAS; Watson et al., 1988). Responses ranged from 0 (very slightly or none of the time) to 4 (extremely; *M* = 0.78, *SD* = 0.60). All continuous covariates were centered at the sample mean.

### Analyses

We fit multilevel models with SAS Proc Mixed to take into account the “nested” data structure, such that the 571 hotel employees were nested within 41 hotels (Raudenbush and Bryk, 2002). Intraclass Correlations (*ICC*) were computed to assess the ratio of the between-organization variability to the total variability in the variable. *ICC*s have been often used to justify the use of multilevel analyses (O'Neill et al., 2009). For example, *ICC*s should be larger than zero to have organization-level variance to be explained. However, large values of *ICC*s (e.g., ≥0.15) also indicate that there is a strong relationship between the data collected from individuals within the same organization (i.e., greater degree of dependence, little unique information; Scherbaum and Ferreter, 2008). Model 1 tested the individual-level effects of job demands and organization-level supervisor's transformational leadership in addition to the effects of all covariates. For psychological job demands, we assessed them only at the individual level, not the organization level, because psychological job demands reflect individual perceptions about their specific job that puts a strain on them; these are likely to vary at the individual level due to different jobs and different individual capacities for those jobs. Thus it does not make sense to aggregate job-level demands to the organizational level. For supervisor's transformational leadership, we used only organization-level (controlling for the individual-level) to assess the individual members' shared perception of the leadership at a hotel. Supervisor's (GM's) leadership style resides at the supervisor level, something that all managers at a hotel share. Thus this is a supervisor-level characteristic, and we wanted to measure a shared view on supervisor's leadership in each organization, which is a more objective measure than individual perceptions. Individual-level predictors were centered at each hotel mean and thus higher scores indicated that hotel managers had higher scores than the other managers within the same hotel. Organization-level predictors were centered at the grand mean, such that higher scores reflected higher averages than the other hotels. In Model 2, we added NWFS and PWFS at both levels. The two-level predictors decomposed variances in each work outcome, such that the effect of organization-level NWFS indicates how working in hotels where the average level of NWFS is higher than the other hotels is associated with more or less exhaustion of the members in the hotels. In Model 3, we added two interaction terms: individual-level NWFS × organization-level PWFS and individual-level NWFS × organization-level NWFS. Note that we estimated fixed effects with no random slopes, because we made hypotheses about the sample-level estimate at each level and did not assume that each employee or each organization had their own slope.

## Results

Table 1 presents descriptive statistics and correlations among key variables. Job characteristics were correlated with work exhaustion and satisfaction in the expected directions. The values of the *ICC* were small, ranging from 0.014 to 0.044, meaning that there was more variability between individuals (managers) than between organizations (hotels). Thus, each manager in a hotel provided unique (not redundant) information of NWFS and PWFS, and work exhaustion and satisfaction, with 1.4–4.4 percent of the variability in the variables were attributable to differences between hotels. In addition, given that our sample included both married/partnered (65%) and single managers, we conducted supplementary difference tests to see whether there are differences by partnered vs. single status in our main variables of interest. There was no difference between married/partnered managers and single managers in the levels of NWFS, PWFS, emotional exhaustion, and job satisfaction.

**Table 1:** **Descriptive statistics and correlations between key variables**.

	***iM (iSD)***	***hM (hSD)***	***Range***	**1**	**2**	**3**	**4**	**5**	**6**
1. Job demands	3.16 (0.52)	−	1.71–4	**0.043**	−^a^	−	−	−	−
2. Supervisor's transformational leadership	3.75 (0.86)	3.69 (0.40)	1.07–5	−0.23	**0.023**	−0.48	0.51	−0.67	0.69
3. Negative work-to-family spillover	3.04 (0.81)	3.08 (0.30)	1–5	0.46	−0.26	**0.025**	−0.37	0.71	−0.57
4. Positive work-to-family spillover	2.87 (0.79)	2.88 (0.31)	1–5	−0.09	0.24	−0.18	**0.014**	−0.61	0.54
5. Emotional exhaustion	2.24 (0.75)	2.31 (0.32)	1–4	0.53	−0.42	0.60	−0.29	**0.044**	−0.79
6. Job Satisfaction	3.58 (1.13)	3.50 (0.50)	1–5	−0.33	0.50	−0.41	0.31	−0.67	**0.044**

*N = 571 hotel managers from 41 hotels; iM and iSD indicate Means and Standard Deviations (SD) at the individual level; hM and hSD indicate Means and Standard Deviations (SD) at the hotel level; All correlations presented at the table were significant at p < 0.05; Diagonals (bold) show Intraclass Correlation Coefficients (ICC = organization-level variance/total variance) of the variable. Numbers below diagonal represents individual-level correlations (based on 571 managers) and numbers above diagonal indicates organization-level correlations (based on 41 hotels)*.

a *Job demands were only assessed at the individual level*.

The results of multilevel models predicting emotional exhaustion and job satisfaction are presented in Tables 2, 3, respectively. Beginning with emotional exhaustion, in Model 1, age, partner employment status, and negative affect were significant covariates for emotional exhaustion. Younger managers (than the sample average = 37.72 years), managers who were living with a non-working spouse/partner (vs. single), and those who had lower negative affectivity reported less emotional exhaustion. Individual-level job demands were positively linked to emotional exhaustion, whereas organization-level supervisor's transformational leadership was negatively linked to exhaustion. Model 1 explained 45% of the variance in emotional exhaustion. Turning to job satisfaction, women and managers who had lower negative affectivity reported higher levels of satisfaction. Individual-level job demands were negatively associated with job satisfaction. Organization-level supervisor's transformational leadership was positively linked to job satisfaction. Job demands and leadership along with covariates explained 37% of the variance in job satisfaction.

**Table 2:** **Results from multilevel models predicting emotional exhaustion**.

	**Model 1**	**Model 2**	**Model 3**
	***B* (*SE*)**	***B (SE)***	***B (SE)***
**Fixed Effects**			
Intercept	2.32 (0.05)^***^	2.29 (0.04)^***^	2.28 (0.05)^***^
**COVARIATES**			
Age	−0.01 (0.00)^***^	−0.01 (0.00)^*^	−0.01 (0.00)^*^
Gender, men (vs. women)	−0.03 (0.05)	0.02 (0.05)	0.02 (0.05)
Number of children	−0.01 (0.03)	0.01 (0.03)	0.01 (0.03)
Living with young children age ≤ 7 (vs. no)	0.17 (0.09)^†^	0.11 (0.08)	0.12 (0.08)
Partner employment			
Partner unemployed (vs. single)	−0.21 (.09)^*^	−0.26 (0.08)^**^	−0.26 (0.08)^**^
Partner employed (vs. single)	−0.00 (0.06)	−0.02 (0.05)	−0.02 (0.05)
Negative affect	0.32 (0.04)^***^	0.18 (0.04)^***^	0.17 (0.04)^***^
**JOB DEMANDS**			
Individual-level	0.55 (0.05)^***^	0.41 (0.05)^***^	0.41 (0.05)^***^
**SUPERVISOR'S TRANSFORMATIONAL LEADERSHIP**			
Individual-level	−0.23 (0.03)^***^	−0.16 (0.03)^***^	−0.16 (0.03)^***^
Organization-level	−0.47 (0.10)^***^	−0.26 (0.10)^*^	−0.26 (0.10)^*^
***NEGATIVE WORK-TO-FAMILY SPILLOVER (NWFS)***			
Individual-level		0.29 (0.04)^***^	0.29 (0.04)^***^
Organization-level		0.40 (0.11)^***^	0.40 (0.11)^***^
***POSITIVE WORK-TO-FAMILY SPILLOVER (PWFS)***			
Individual-level		−0.14 (0.03)^***^	−0.14 (0.03)^***^
Organization-level		−0.34 (0.12)^**^	−0.34 (0.12)^**^
***INTERACTIONS BETWEEN INDIVIDUAL-LEVEL NWFS AND ORGANIZATION-LEVEL PWFS/NWFS***			
Individual NWFS × Organization PWFS			−0.17 (0.12)
Individual NWFS × Organization NWFS			0.06 (0.12)
**Random Effects**			
Organization-level variance	0.009 (0.01)	0.003 (0.01)	0.004 (0.01)
Individual-level variance	0.310 (0.02)^***^	0.259 (0.02)^***^	0.258 (0.02)^***^
Pseudo *R^2^*	0.448	0.551	0.552

*N = 571 hotel managers from 41 hotels; For categorical predictors, reference group (coded as 0) is in parenthesis*.

† p < 0.10,

* p < 0.05,

** p < 0.01,

*** *p < 0.001*.

**Table 3:** **Results from multilevel models predicting job satisfaction**.

	**Model 1**	**Model 2**	**Model 3**
	***B* (*SE*)**	***B (SE)***	***B (SE)***
**Fixed Effects**			
Intercept	3.55 (0.08)^***^	3.58 (0.08)^***^	3.59 (0.08)^***^
**COVARIATES**			
Age	0.01 (0.00)	0.00 (0.00)	0.00 (0.00)
Gender, men (vs. women)	−0.22 (0.08)^**^	−0.28 (0.08)^***^	−0.28 (0.08)^***^
Number of children	0.05 (0.05)	0.03 (0.05)	0.03 (0.05)
Living with young children age ≤ 7 (vs. no)	−0.21 (0.15)	−0.14 (0.14)	−0.15 (0.14)
Partner employment			
Partner unemployed (vs. single)	0.23 (0.15)	0.28 (0.14)^*^	0.27 (0.14)^†^
Partner employed (vs. single)	0.04 (0.09)	0.07 (0.09)	0.06 (0.09)
Negative affect	−0.48 (0.07)^***^	−0.36 (0.07)^***^	−0.35 (0.07)^***^
**JOB DEMANDS**			
Individual-level	−0.37 (0.08)^***^	−0.27 (0.08)^**^	−0.27 (0.08)^**^
**SUPERVISOR'S TRANSFORMATIONAL LEADERSHIP**			
Individual-level	0.50 (0.05)^***^	0.43 (0.05)^***^	0.43 (0.05)^***^
Organization-level	0.71 (0.16)^***^	0.52 (0.18)^**^	0.52 (0.19)^**^
***NEGATIVE WORK-TO-FAMILY SPILLOVER (NWFS)***			
Individual-level		−0.22 (0.06)^***^	−0.21 (0.06)^***^
Organization-level		−0.25 (0.20)	−0.26 (0.20)
***POSITIVE WORK-TO-FAMILY SPILLOVER (PWFS)***			
Individual-level		0.25 (0.05)^***^	0.25 (0.05)^***^
Organization-level		0.41 (0.22)^†^	0.40 (0.22)^†^
***INTERACTIONS BETWEEN INDIVIDUAL-LEVEL NWFS AND ORGANIZATION-LEVEL PWFS/NWFS***			
Individual NWFS × Organization PWFS			0.44 (0.21)^*^
Individual NWFS × Organization NWFS			−0.03 (0.20)
**Random Effects**			
Organization-level variance	0.034 (0.02)^†^	0.030 (0.02)^†^	0.030 (0.02)^†^
Individual-level variance	0.784 (0.05)^***^	0.727 (0.05)^***^	0.723 (0.05)^***^
Pseudo **R^2^**	0.366	0.419	0.424

*N = 571 hotel managers from 41 hotels; For categorical predictors, reference group (coded as 0) is in parenthesis*.

† p < 0.10,

* p < 0.05,

** p < 0.01,

*** *p < 0.001*.

After adjusting for these effects, Model 2 tested whether NWFS and PWFS at both the individual and organizational levels were significantly associated with emotional exhaustion and job satisfaction. At the individual-level, managers who experienced higher NWFS than the other managers in the same hotel reported more exhaustion, but those with higher PWFS reported less exhaustion (H1 was supported). Moreover, managers who experienced higher NWFS reported lower satisfaction, whereas those with higher PWFS reported higher satisfaction (H2 was supported). At the organization-level, working in hotels where the average level of NWFS was higher than the other hotels was associated with feeling higher emotional exhaustion, whereas working in hotels where the PWFS was higher was associated with feeling lower emotional exhaustion (H3 was supported). However, organization-level NWFS and PWFS were not significantly associated with the members' job satisfaction (H4 was not supported). The inclusion of NWFS and PWFS contributed to an additional 11% of the variance in emotional exhaustion and an additional 5% variance in job satisfaction which were not accounted by job demands and supervisor's transformational leadership.

**Figure 1:**
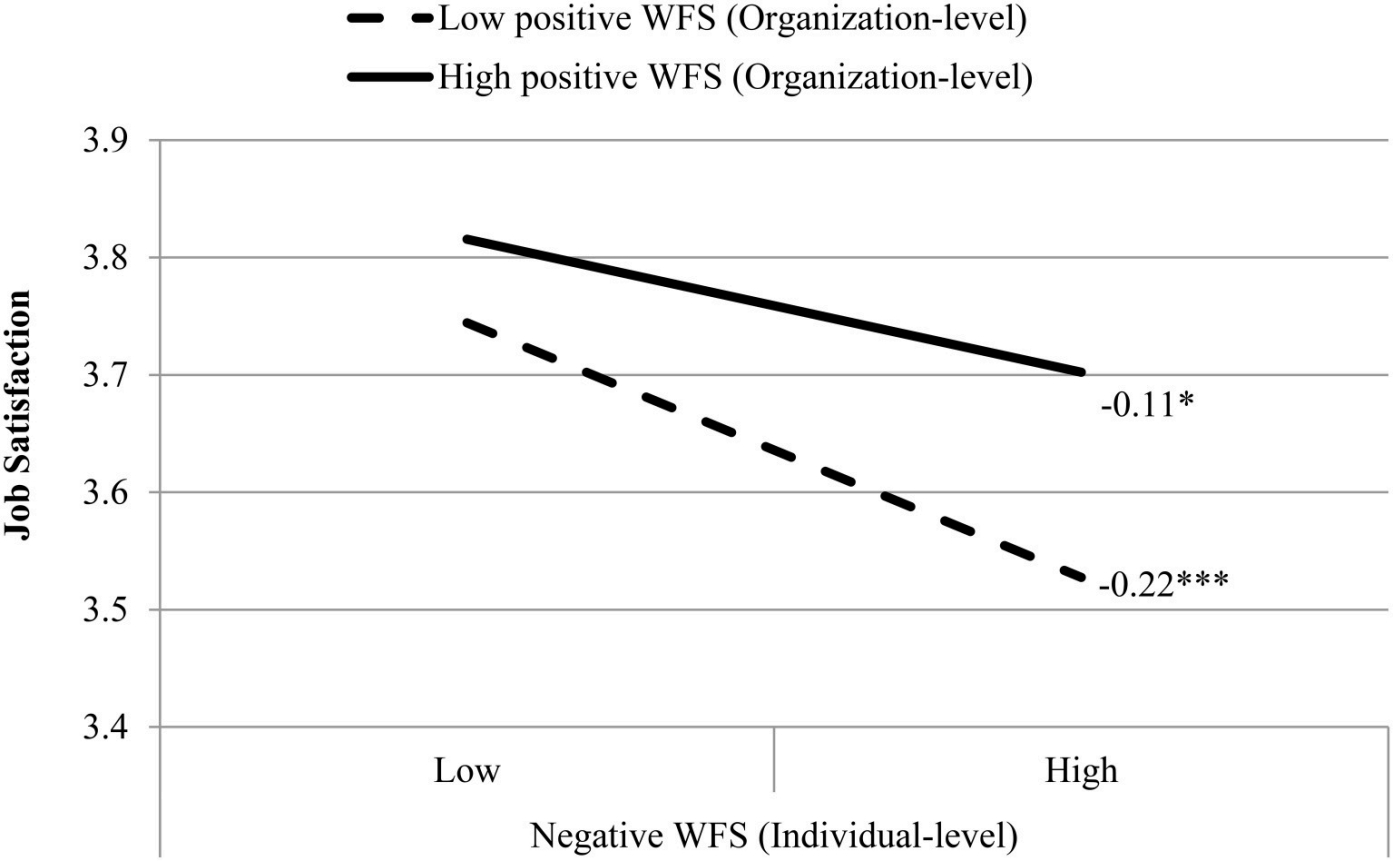
**Interactive effect between organization-level positive work-to-family spillover and individual-level negative work-to-family spillover on hotel managers' job satisfaction**. *N* = 571 from 41 hotels. WFS, Work-to-Family Spillover; Low and high means standard deviation below and above the sample mean, respectively. A half standard deviation was chosen in order to have an adequate sample size for conducting the necessary statistical tests across the cells, following the common practice in previous research (e.g., Almeida et al., 2015). ^*^ p < 0.05, ^***^ p < 0.001.

Model 3 included interactions between individual-level NWFS and organization-level PWFS and NWFS, in addition to all variables from the prior model. However, none of the interactions were significantly associated with emotional exhaustion (H5 and H6 were not supported). However, the interaction between individual-level NWFS and organization-level PWFS was significantly associated with job satisfaction. Figure 1 shows that, for managers who experienced higher NWFS, the negative link between NWFS and job satisfaction was weaker when they worked in hotels where the average level of PWFS was higher, compared to when they worked in hotels where PWFS was lower (H7 was supported). The interaction between individual-level NWFS and organization-level NWFS was not significantly associated with job satisfaction (H8 was not supported).

## Discussion

Drawing upon the conservation of resources theory (Hobfòll, 1989, 2001), this study examined the unique and interactive associations of negative and positive WFS with hotel managers' work exhaustion and satisfaction, independent of psychological job demands and supervisor's leadership style. Guided by the levels of analysis framework (Kozlowski and Klein, 2000), we examined the associations at the organizational aggregated level, in addition to individual manager level. Results generally supported our hypotheses. NWFS and PWFS were uniquely associated with emotional exhaustion and job satisfaction, even after adjusting for how many demands and how much transformational leadership managers had in their work context. There were also significant organizational climate effects of NWFS and PWFS on emotional exhaustion. Moreover, there was a cross-level interactive effect of NWFS and PWFS, such that a high level of organization-level PWFS buffered the negative effect of individual-level NWFS on job satisfaction. This study moves beyond a focus on traditional job characteristics, toward considering individual and organizational experiences in the work-family interface as important predictors of work-related outcomes. The unique effects of individual WFS on hotel managers' emotional exhaustion and job satisfaction demonstrate the need to provide workplace policies that target work and family interconnections in hotel employees' lives, which may, in turn, increase their work productivity (Anttonen and Vainio, 2010). The results may also be broadly applicable to a range of occupations (other than hotel managers) where high NWFS is a concern (Nomaguchi, 2009).

Our findings clearly indicate that NWFS and PWFS are shared with coworkers, and thus signify a specific component of organizational work-family climate. Being part of a hotel with higher NWFS and lower PWFS was associated with higher emotional exhaustion for managers at the hotel. A number of studies have documented the significance of WFS—primarily NWFS—for employee well-being at the individual-level; however, little research has examined the organization-level influence of WFS (but see van Emmerik and Peeters, 2009; Moen et al., 2015, for exceptions). Our findings contribute to understanding of an additional link: Experience of NWFS is shared within the same organization, such that working in an organization where many coworkers experience high NWFS can lead to feeling exhausted for individual members at the organization. This finding is in line with the levels of analysis framework (Kozlowski and Klein, 2000) in that working in organizations where the overall NWFS is high may drain psychological resources of individual members at the organization. Again, these organization-level associations were found after controlling for individual-level WFS, meaning that organizational influence of WFS on emotional exhaustion exists regardless of individual managers' own experiences of WFS. Note, however, that we did not find significant main effects of organizational WFS on job satisfaction; it seems that job satisfaction is less likely to be affected by the shared experiences of WFS between coworkers. Unlike emotional exhaustion, job satisfaction may be more related to a personal evaluation about his/her job (e.g., “You are satisfied with your present job situation”). Therefore, whether coworkers in the same hotel experience NWFS or PWFS may not be significantly associated with the hotel managers' own job satisfaction overall. However, we found in the cross-level interactive effect that organizational PWFS becomes important for individual managers' job satisfaction when they experience high levels of NWFS.

The cross-level interaction effect of NWFS and PWFS on job satisfaction showed that working in an organization where the average level of PWFS is high can buffer the negative effect of individual-level NWFS. This finding supports the COR theory (Hobfòll, 2001) that highlights the saliency of higher-level support under the individual experience of stress. To the best of our knowledge, this is the first study that shows the importance of a positive work-family climate in terms of its buffering effect (but see Feierabend et al., 2011, for its main effect on employee outcomes). Considering that there is an increasing trend of NWFS with increased competition and 24–7 operations in a range of occupations (Nomaguchi, 2009), focusing on PWFS may help protect or improve employees' satisfaction and attitudes toward their work. Note also that we did not find a significant interactive association between organization-level NWFS and individual-level NWFS on emotional exhaustion or job satisfaction. Perhaps, NWFS on an individual level is sufficient to impart damage on work-related well-being outcomes, regardless of how much anyone else is suffering NWFS. This idea is consistent with the COR perspective (Hobfòll, 1989) that stressors narrow one's focus to be self-protective; thus those with higher NWFS may not be able to see others' pain or respond to it. This assumption, however, needs to be empirically tested in future research.

In terms of practical implications, we suggest implementing workplace programs that target increasing PWFS in the organization. Increasing organizational PWFS requires increasing each employee's PWFS. However, rather than attributing to employee-level responsibility, organizations could make workplace-level efforts to increase their employees' PWFS. For example, having facilitated discussions with employees to share strategies to increase PWFS or a message board to post PWFS experiences may increase chances for individual-level PWFS. Moreover, considering that organizational PWFS reflects role modeling and sharing processes between coworkers, educating leaders and senior managers to give more positive feedback to their team members and show more family-supportive behavior (Hammer et al., 2011; Bray et al., 2013; Kossek et al., 2014) may create more positive work episodes that may “spill over” to the home and facilitate performing family roles. Increased individual-level PWFS may, in turn, create more resources and support in the workplace available by coworkers that can protect the job satisfaction of employees who suffer from high NWFS, as evidenced in this study. Most workplace intervention programs have focused on reducing a negative aspect of work at individual level, such as decreasing NWFS experienced by employees (Kelly et al., 2011, 2014). However, future workplace efforts could move toward increasing PWFS of their employees to create a positive work environment.

## Limitations and future directions

The current study has limitations. First, we used self-reports to measure WFS and well-being, which poses a potential risk for a common-method bias (Podsakoff et al., 2003). Although we addressed this issue in part, by incorporating organization-level ratings by aggregating multiple assessments from several managers within each hotel, individual-level effects might still be inflated by common-method bias. To address this problem, future research should incorporate more objective data (e.g., biomarkers, job performance evaluated by organizations). Second, Cronbach's alpha for PWFS in the present study was less than desirable (0.61). This raises an issue regarding the reliability of the scale, which may lead to limited power to find effects. Future research should refine measurement of this construct. Third, the cross-sectional data constrain our ability to identify any causality. Although our statistical models imply that NWFS and PWFS predict emotional exhaustion and job satisfaction, our design does not rule out reverse causality. In other words, it is possible that one feels dissatisfied or exhausted at work, and this creates negative WFS. There is likely a spiraling effect, as proposed by COR (Hobfòll, 1989), over time. Future work could use a more rigorous assessment of spillover, such as separately measuring stressors at work and mood and behaviors at home across time. Then changes in mood or behaviors at home as a function of stressors at work may indicate the degree of spillover (e.g., Judge and Ilies, 2004). Using this within-person slope of spillover, future analyses could examine how spillover predicts work- and family-related well-being outcomes. Lastly, we used a sample of hotel managers in the U.S. context, and thus our findings may not generalize to the population of employees in other industries or hotel managers in other cultural contexts.

In conclusion, this study suggests that the experience of high NWFS and low PWFS is one of the main reasons why hotel managers become exhausted or dissatisfied with their job. Our results illuminate the importance of organization-level PWFS. Future research should continue to examine the implications of organization- and individual-level NWFS and PWFS with diverse industry samples and their long-term effects on employees and family members. Although it may not be easy to change the trend of increasing NWFS, such efforts may help understand how PWFS is protective of employee well-being and their family relationships from the adverse effects of NWFS.

## Author contributions

SL contributed to the design, draft and revision of the current manuscript. She also analyzed and interpreted data. KD contributed to the acquisition of data, revising the current manuscript, and investigation of the accuracy of the work. CN contributed to the initial design and draft of this study and the revision of the current manuscript. She also contributed to the initial analyses and interpretation of data. AG contributed to design of research questions and strengthening theoretical arguments. CL contributed to the initial design, analyses and interpretation of data. DA contributed to the acquisition of data, design of this study, revising the manuscript, ensuring the accuracy of work, and final approval of the version to be submitted.

## Funding

This research was conducted as part of the Work, Family, and Health Network, which is funded by the National Institute of Child Health and Human Development (U01 HD051217-03). We also thank Alfred P. Sloan Foundation (2004-12-4), The Penn State General Clinical Research Center (NIH Grant M01-RR-10732), Johnson & Johnson Consumer and Personal Products Worldwide and the PSU College of Health and Human Development, and the PSU Child, Youth, and Families Consortium part of the Social Science Research Institute for providing additional support for this research. Finally, we would like to thank the hotel employees and their families for their participation.

### Conflict of interest statement

The authors declare that the research was conducted in the absence of any commercial or financial relationships that could be construed as a potential conflict of interest.
